# Autoantibodies as predictors of progression to rheumatoid arthritis: a systematic review and meta-analysis

**DOI:** 10.1136/rmdopen-2025-006368

**Published:** 2026-02-11

**Authors:** Sumera Qureshi, Maryam Adas, Phoebe J Cope, Hassan Mahfouz, Katie Bechman, Kevin D Deane, Hani El-Gabalawy, Paul Emery, Axel Finckh, Benoît Thomas P Gilbert, V Michael Holers, John D Isaacs, Alf Kastbom, Kulveer Mankia, Ted R Mikuls, Arthur G Pratt, Juergen Rech, Mark D Russell, Georg Schett, Leendert A Trouw, Carl Turesson, Marian H van Beers-Tas, Annette H M van der Helm-van Mil, Dirkjan van Schaardenburg, Hanna W van Steenbergen, René E M Toes, Andrew P Cope, James Galloway, Sam Norton

**Affiliations:** 1 Centre for Rheumatic Diseases, King’s College London, London, UK; 2 King’s College London, London, UK; 3 Division of Rheumatology, University of Colorado Anschutz Medical Campus, Aurora, Colorado, USA; 4 Arthritis Centre, Department of Internal Medicine, University of Manitoba, Winnipeg, Manitoba, Canada; 5 Leeds Institute of Rheumatic and Musculoskeletal Medicine, University of Leeds and NIHR Leeds Biomedical Research Centre, Leeds Teaching Hospitals NHS Trust, Chapel Allerton Hospital, Leeds, UK; 6 Division of Rheumatology, Geneva University Hospitals, Geneva, Switzerland; 7 NIHR Newcastle Biomedical Research Centre at Newcastle-upon-Tyne Hospitals NHS Foundation Trust and Newcastle University, Newcastle-upon-Tyne, UK; 8 Department of Biomedical and Clinical Sciences, Linköping University, Linköping, Sweden; 9 Department of Internal Medicine, Division of Rheumatology, College of Medicine, University of Nebraska Medical Center; Medicine, Veterans Affairs Nebraska-Western Iowa Health Care System, Omaha, Nebraska, USA; 10 Newcastle Biomedical Research Centre at Newcastle-upon-Tyne Hospitals NHS Foundation Trust and Newcastle University, Newcastle-upon-Tyne, UK; 11 Department of Internal Medicine 3 - Rheumatology and Immunology, Friedrich-Alexander University Erlangen-Nürnberg and Universitätsklinikum Erlangen, Erlangen, Germany; 12 Deutsches Zentrum für Immuntherapie, Friedrich Alexander University Erlangen-Nuremberg and Universitätsklinikum Erlangen, Erlangen, Germany; 13 NIHR Advanced Fellow, Centre for Rheumatic Diseases, King’s College, London, UK; 14 Department of Immunology, Leiden University Medical Center, Leiden, The Netherlands; 15 Department of Clinical Sciences, Lund University, Malmö, Sweden; 16 Department of Rheumatology, Amsterdam Rheumatology and Immunology Center, Amsterdam, The Netherlands; 17 Department of Rheumatology, Leiden University Medical Center, Leiden, The Netherlands; 18 Reade Center for Rheumatology and Rehabilitation, Department of Clinical Immunology and Rheumatology, Amsterdam UMC, University of Amsterdam, Amsterdam, The Netherlands; 19 Versus Arthritis Professor of Rheumatology, Centre for Rheumatic Diseases, King’s College London, London, UK; 20 Professor of Rheumatology, Centre for Rheumatic Diseases, King’s College London, London, UK; 21 Professor of Medical Statistics & Applied Health Research, Centre for Rheumatic Diseases, King’s College London, London, UK

**Keywords:** Arthritis, Rheumatoid, Anti-Citrullinated Protein Antibodies, Rheumatoid Factor, Epidemiology

## Abstract

**Background:**

The aim of this systematic review and meta-analysis was to examine autoantibody positive individuals and define: (a) the relative risk of rheumatoid arthritis (RA) compared with autoantibody negative individuals and (b) the cumulative incidence of RA over time, in different populations.

**Methods:**

A systematic literature search was performed in August 2025. Retrospective case control studies of individuals with RA and healthy matched controls were identified and relative risk of RA was calculated. Prospective observational studies or randomised controlled trials of autoantibody positive individuals were identified and pooled survival models were used to estimate cumulative incidence of progression to arthritis.

**Results:**

293 full-text articles fulfilled screening criteria and 26 were eligible for meta-analysis. The presence of serum autoantibodies confers between a 3.1--19.3-fold increase risk of developing RA. Prospective studies of anticyclic citrullinated peptide 2 (CCP2) or CCP3 positive individuals were grouped according to additional criteria; presence of arthralgia, presence/absence of immunoglobulin M rheumatoid factor (IgM-RF) and first-degree or second-degree relatives with RA. The estimated cumulative incidence of RA at 12 months was highest for CCP2 positive individuals with arthralgia and IgM-RF, at 35.2% (95% CI 29.3% to 41.2%).

**Conclusion:**

A considerable number of autoantibodies have been examined as predictors for RA; however, the fastest rate of progression to RA in this study occurred in those with CCP2 and IgM-RF in combination with arthralgia. Importantly, the risk of developing RA changes over time for individuals with arthralgia, and they are at the highest risk of progression within the first 24 months of follow-up.

**PROSPERO registration number:**

CRD42021231245.

**WHAT IS ALREADY KNOWN ON THIS TOPIC**

- The risk of developing rheumatoid arthritis (RA) is increased by the presence of autoantibodies in healthy individuals; however, the rate of progression to RA within these populations is highly variable. At the time of the initiation of this study, prospective observational cohorts and randomised controlled trials (RCTs) have used varied combinations of serology, imaging and symptoms to predict progression to RA and there was no consensus on the optimal use of serology.

**WHAT THIS STUDY ADDS**

- In the largest meta-analysis to date of over 2000 individuals, we found the risk of RA is increased 19-fold in individuals with anticitrullinated protein antibodies (ACPAs), compared to ACPA negative individuals. Using innovative meta-analytical techniques to synthesise and extract patient level data, we have been able to provide more precise risk estimates for ACPA positive individuals at risk of RA. When these individuals were followed up for up to 5 years, the fastest rate of progression to RA occurred in the presence of serum citrullinated autoantibodies generation 2 (CCP2) and IgM-RF in combination with arthralgia. Importantly, this risk changes over time—the highest risk is within the first 2 years and markedly decreases after this period.

**HOW THIS STUDY MIGHT AFFECT RESEARCH, PRACTICE OR POLICY**

- In order to stratify individuals at highest risk of progression to RA within 2 years, the presence of arthralgia, IgM-RF and CCP2 should be assessed. This can help to inform both future clinical trial design targeting prevention and for practising clinical rheumatologists. We suggest that these individuals should be most closely monitored in the first 24 months, after which point a risk remains, but is markedly reduced. Importantly, over the follow-up period of 5 years, no cohort had 100% progression to RA. Further research to characterise these subsets of ‘resilient’ individuals within high-risk groups is important, and recognition of this in clinical practice is key to prevent overtreatment.

## Introduction

 Serum rheumatoid factor (RF) and anti-citrullinated protein antibodies (ACPAs) are important diagnostic tools for rheumatoid arthritis (RA) and their detection underpins one of the four domains of the 2010 American College Rheumatology (ACR)/European League Against Rheumatism (EULAR) classification criteria for RA^1^. The use of autoantibodies as predictive tools for the development of RA was first hypothesised following early observations from prospective cohorts^2^ and examination of stored sera in medical biobanks^3^. In some cases, individuals with RA were found to have RF and ACPA present up to 10 years prior to the onset of disease.^4 5^ These findings led to the recognition of a seropositive ‘at-risk’ phase in the evolution of the disease, distinct from genetic^6^ and environmental risk factors.^7^ The occurrence of joint symptoms, and more recently, subclinical imaging changes has been identified as further ‘at-risk’ phases in the development of RA.^7 8^

Using this paradigm, multiple prospective cohort studies have since been established in the UK, Europe and the Americas. Using clinical and population screening approaches, precise titres or combinations of autoantibodies have been used to identify and classify individuals as having increased risk of progression to RA.^9 10^ Since 2010, several randomised controlled trials (RCTs) have sought to ‘intercept’ progression and delay or prevent the clinical onset of disease, adopting heterogeneous autoantibody, genetic, clinical or imaging criteria.^11^,^15^ Recently, two of these RCTs have demonstrated statistically significant delayed progression to RA in subsets of individuals with autoantibodies in combination with clinical symptoms and/or evidence of subclinical inflammation by imaging at enrolment.^16 17^

This has led to a concerted effort to homogenise the design and recruitment of at-risk individuals into these studies. A 2017 EULAR taskforce used clinical expertise to select five features of the history and two examination findings to define arthralgia at risk for RA, termed ‘clinically suspect arthralgia’^18^. A later taskforce also listed ten points to consider for investigators designing observational studies and clinical trials.^19^ More recently, in May 2025, a joint EULAR/ACR taskforce reported a validated risk stratification tool that used clinical, serological and imaging variables to predict development of RA in individuals with arthralgia who present to healthcare settings.^20^ ACPA and RF were selected in this model, alongside four other clinical, serological and imaging parameters to define more homogeneous risk groups for future prevention trials.

The objective of this systematic review and meta-analysis was to examine the literature in at-risk cohorts to (a) estimate the overall relative risk of RA in autoantibody positive individuals compared with autoantibody negative individuals and (b) define the cumulative incidence of RA within these autoantibody positive individuals over time, in different at-risk populations

## Methods

### Search strategy and selection criteria

We performed a systematic literature review and meta-analysis. Two reviewers (SQ and PJC) independently undertook the literature search, article screening and study selection, with involvement of APC to resolve discrepancies. MEDLINE, Embase, Global Health (via OVID) and SCOPUS were searched from inception to August 2023, with English language restrictions using combinations of search terms: ‘rheumatoid arthritis’, ‘autoantibodies’ ‘pre-clinical’ and their synonyms (see online supplemental information for the full list of search terms used). The search terms and strategy for MEDLINE (via Ovid) were adapted for other databases. The reference lists of eligible studies were also searched for additional eligible studies. A search was repeated in August 2025 for studies from August 2023 to August 2025 by SQ, HM and PJC. This systematic review and meta-analysis followed the Preferred Reporting Items for Systematic Reviews and Meta-analyses. The study protocol was registered at PROSPERO (registration ID: CRD42021231245).

Studies were selected if they evaluated the presence of autoantibodies in individuals aged 18 years or over, who did not have clinically apparent arthritis at baseline. Autoantibodies included, but were not limited to, RF, autoantibodies to modified protein antigens including ACPA and antibodies to carbamylated proteins (anti-CarP). Thresholds for positivity were informed by the upper limit of normal defined by each specific assay and results using specific antibody isotype detection methods were included. Eligible studies for inclusion were prospective cohort studies, retrospective cohort studies, case-control studies, RCTs and case series reporting at least 10 patients. Case reports or case series with fewer than 10 patients were excluded. Studies were excluded if participants had evidence of clinical joint swelling at baseline, early/undifferentiated RA, palindromic RA or were receiving any medication for RA during or prior to the period of follow-up. Outcomes of interest were the development of RA, as defined by the 1987 ACR or 2010 ACR/EULAR classification criteria for RA or clinical confirmation by a trained observer of at least one clinically swollen joint not attributable to other rheumatic diseases. As all individuals were positive for autoantibodies, this outcome was by-definition risk of seropositive RA.

### Data analysis

Study characteristics extracted included study type, participant numbers, demographics, autoantibody analysed, other cohort selection criteria (eg, imaging, symptoms or family history (FH) of RA) and outcome measures as described above. Where prospective observational cohorts included individuals who were not autoantibody positive at baseline, data were extracted only for autoantibody positive individuals. For RCTs, data from placebo arms were extracted. If an eligible cohort or paper was identified but datasets were either incomplete or not published, primary authors were contacted inviting them to provide further information. Discrepancies arising between reviewers during study selection or data extraction were resolved through consensus discussion, with involvement of APC.

Studies were stratified by (a) study type and (b) autoantibody. To avoid bias and to examine for duplication of data, all eligible studies were extracted. For the meta-analysis, all studies reporting on the same autoantibody and cohort were then examined and one study per cohort was selected, prioritising either the most complete dataset or the largest number of participants examined. If a study explicitly stated they had used a distinct group of patients from a cohort (eg, individuals recruited later) they were deemed suitable for pooling with other individuals from the same cohort. Finally, where there was incomplete data reporting of cohorts, lead investigators were contacted for confirmation that duplicate data had not been analysed.

SQ and HM assessed risk of bias for case control and cohort studies independently. The risk of bias was assessed using the Newcastle-Ottawa Scale Quality Assessment tool.^21^ For cohort studies in our analysis, there was no comparability domain applicable. SQ and MA assessed risk of bias for RCTs together across different domains according to the Cochrane Collaboration’s Risk of Bias 2 tool.^22^ Statistical evidence of between-study heterogeneity was examined using the I^2^ statistic. The robustness of pooled estimates was assessed via a leave-one-out sensitivity analysis. A study was deemed influential if the pooled estimate without it was not within the 95% CIs of the overall pooled estimate. Publication bias was assessed via visual inspection of funnel plots and Egger’s test. If small sample effects bias was identified, the precision-effect test and precision-effect estimate with standard errors (PET-PEESE) approach was used to provide a bias-adjusted pooled estimate.

To calculate the relative risk, the number of RA patients with autoantibodies detected prior to the onset of disease was compared with the number of matched individuals without RA who were also autoantibody positive. For the individuals selected as controls in these studies, data on joint symptoms or other risk factors for autoantibody positivity, such as infection, were not reported and thus could not be evaluated. This approach (eg, unadjusted risk ratios) was selected given the heterogeneity in approaches individual studies used, and variables were adjusted for as potential confounders, to ensure comparability of the estimates across studies. Using random-effects models, a pooled risk ratio with corresponding 95% CIs was calculated. The random-effects model was selected a priori due to the anticipated heterogeneity of the included studies. A fixed continuity correction of 0.5 was applied to studies with zero events.

For each study, progression to RA was as per the study authors’ own definition, which was typically 2010 ACR/EULAR classification criteria or clinical confirmation of at least one clinically swollen joint as described above. The risk of progression to RA in prospective studies was examined by calculating cumulative incidence of RA, as follows:

1. DigitiseIt software application (http://www.digitiseit.de/) extracted co-ordinates from published Kaplan-Meier (KM) survival/failure graphs.^23^
2. Number of individuals at risk at the start of follow-up was extracted. This was defined as per the study authors, typically point of referral to rheumatology for observational studies and randomisation for clinical trials.
3. Numbers at risk at regular time intervals during follow-up (usually presented as the risk table) and/or total number of individuals who developed outcome of interest were extracted to improve accuracy of the digitally reconstructed KM survival graph.
4. Survival curves were reconstructed using the ‘ipdfc’ command in STATA^24^ and visually inspected to compare accuracy.
5. Pooling of data was applied using a two-level mixed-effects parametric survival model with a shared frailty accounting for nesting of people with studies. For all models, an exponential distribution was selected as the best fitting survival distribution compared with Weibull, log-logistic, log-normal and gamma, based on the Bayesian Information Criterion statistic.

All analyses were performed using Stata (V.17.0/IC; StataCorp). Two-tailed p<0.05 was considered statistically significant.

### Role of the funding source

Sumera Qureshi’s clinical research fellowship was funded by Bristol Myers Squibb who had no part in the design, analysis or writing of the manuscript.

## Results

**Figure 1:**
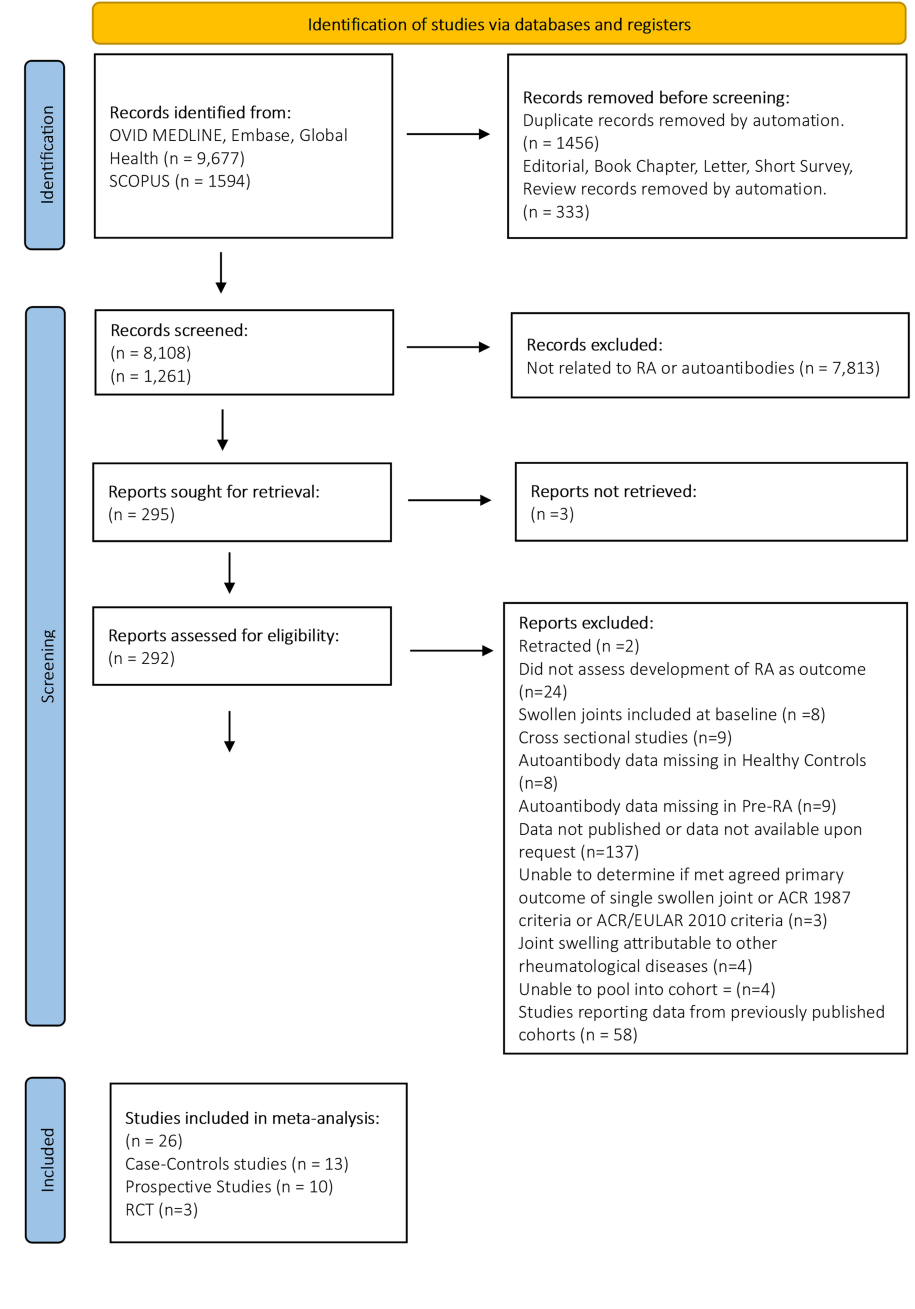
Preferred Reporting Items for Systematic Reviews and Meta-Analyses 2020 flow diagram. ACR, American College of Rheumatology; EULAR, European League Against Rheumatism; RA, rheumatoid arthritis; RCT, randomised controlled trial.

The systematic literature search identified 8108 unique studies. After screening the title and abstracts, 293 full text articles were assessed and 26 were eligible for meta-analysis (figure 1).

To calculate the relative risk of developing RA, 13 retrospective case control studies were analysed. A total of 1242 individuals with serum available prior to the onset of RA were tested for autoantibodies, as well as 2572 matched healthy controls. The mean age ranged from 37 years to 58 years and females comprised between 48–100% of the cohorts. Studies were published between 2003 to 2022. Six autoantibodies were identified and eligible for pooling: CCP2, CCP3, IgA-RF, IgG-RF, IgM-RF and anti-CarP. The CCP2+IgM RF combination was evaluated, whereas other autoantibody combinations had too few studies to permit meta-analysis.

To calculate the risk of progression to RA, 10 prospective observational cohorts and three RCTs were identified, with a total of 1180 autoantibody positive individuals without joint swelling followed up for the development of RA for between 2 years and 12 years. A total of 1005 autoantibody positive individuals were identified from prospective observational cohorts, with a mean age ranging from 44 years to 52 years and females comprising between 72–83% of the cohorts. Cohorts were published between 2010 and 2023. A total of 175 autoantibody positive individuals were recruited to the placebo arm of RCTs, with a mean age ranging from 46 years to 49 years and females comprising between 66–81% of the cohorts. RCTs were published between 2010 and 2024. Three autoantibodies were identified as eligible for pooling.

The characteristics of all included studies are summarised in tables1  2; and 11 studies utilised additional data provided by study authors. All RCTs were deemed to be at low risk of bias. All other studies had potential sources of bias (see online supplemental tables 1–3).

**Table 1:** Case control studies

Source region (country)	Author	Data provided by authors	Study cohort	Cohort (n)	Sex (% female)	Age (mean)	Ever smokers (%)	Outcome	Autoantibody extracted and pooled	Threshold for positivity
USA	Mikuls *et al*^43^	Yes	Military cohort - DODSR and WRMC: ‘*Notably, none of these 346 cases were included in an earlier evaluation of 83 individuals with RA in the DoDSR by our group’*	Cases (212)	48	37	32	1987 ACR classification criteria	CCP2, IgM-RF	≥1 ULN
				Case control Comparator Population (207)	48	37	23	No RA or inflammatory Arthritis	CCP2, IgM-RF	As above
	Kelmenson *et al*^28^	No	Military cohort - DODSR and WRMC: ‘*Notably, none of these 346 cases were included in an earlier evaluation of 83 individuals with RA in the DoDSR by our group’*	Cases (214)	48	37	32	1987 ACR classification criteria or Rheumatologist diagnosed	IgA-RF, IgG-RF	Cut-off defined as levels present in<2% of controls
				Case control Comparator Population (210)	48	37	23	‘And matched controls obtained from the US military.’	IgA-RF, IgG-RF	As above
	Fetchner *et al*^45^	No	Military cohort - DODSR and WRMC	Cases (83)	61	39.9	*	1987 ACR classification criteria or Rheumatologist diagnosed	IgA-RF, IgG-RF, CCP2	Cut-off defined as levels present in<5% of controls or≥1 ULN
				Case control Comparator Population (83)	61	40.0	*	‘Without RA’	IgA-RF, IgG-RF, CCP2	As above
	Gan *et al*^46^	No	Military cohort - DODSR and WRMC	Cases (76)	40.8	39.8	*	1987 ACR classification criteria or Rheumatologist diagnosed	CarP. IgM-RF	Positivity for all antibodies was determined as ≥2 SD of log-transformed means from controls.
				Case control Comparator Population (41)	39	40.6	*	‘Without RA’	CarP. IgM-RF	As above
	Arkema *et al*^47^	No	Nurses registry - Nurses’ Health Study I and Nurses’ Health Study II	Cases (192)	100	52.3	56.8	1987 American College of Rheumatology classification criteria	CCP2	≥1 ULN
				Case control Comparator Population (567)	100	52.4	50.8	No RA or connective tissue disease (self-reported)	CCP2	As above
Netherlands	Shi *et al*^48^	Yes	Sanquin Blood Bank Northwest Region (formerly Red Cross Blood Bank) in Amsterdam	Cases (79)	62	51.4	*	1987 American College of Rheumatology classification criteria	CCP2, CarP	Positivity for all antibodies was determined as ≥2 SD of log-transformed means from controls.
				Case control Comparator Population (141)	Matched	‘+ or - 3 years’	*		CCP2, CarP	As above
Sweden	Sundstrom *et al*^49^	No	Västerbotten Intervention Programme and Department of Rheumatology, University Hospital, Umeå, Sweden	Cases (355)	70	50	62.8	1987 ACR criteria for a diagnosis of RA	CCP2	Missing
				Case control Comparator Population (1007)	70	50	40.8	Missing	CCP2	Missing
	Brink *et al*^50^	Yes	Medical Biobank and the Maternity cohort, Department of Rheumatology, University Hospital in Umeå	Cases (252)	77	52 (median age)	65	1987 American College of Rheumatology classification criteria	CarP	256.07 arbitrary units/mL
				Case control Comparator Population (197)	75	50 (median age)	50	Missing	CarP	As above
	Rantapaa *et al*^5^	No	The Northern Sweden Health and Disease Study cohort; the Maternity cohort of Northern Sweden, Department of Rheumatology, University Hospital in Umeå	Cases (83)	83	54; 29	62.5	1987 American College of Rheumatology classification criteria	IgA-RF, IgG-RF	95th percentile value of the controls as the cut-off point
				Case control Comparator Population (382)	85	55; 30	46.4	Missing	IgA-RF, IgG-RF	As above
	Turesson *et al*^51^	Yes	Malmö Diet Cancer Study	Cases (169)	79	58.1	66.5	1987 American College of Rheumatology classification criteria	CCP2, IgM-RF	≥1 ULN
				Case control Comparator Population (168)	79	58.3	59.3	Alive and free from RA when the index person was diagnosed with RA	CCP2, IgM-RF	≥1 ULN
Norway	Jorgensen *et al*^4^	No	Blood donors in Oslo, Norway, collected between 1973 and 2000, in the JANUS Serum Bank	Cases (49)	61%	41	*	ACR 1987 classification criteria	CCP, IgM-RF	≥1 ULN, 17 IU/mL
				Case control Comparator Population (245)	63%	‘Matched’	*	‘Without RA’	CCP, IgM-RF	As above
Multicentre	Fisher *et al*^52^	No	EPIC	Cases (103)	*	*	*	1987 or 2010 ACR/EULAR criteria	CCP2, IgM-RF	≥1 ULN or agglutination at a dilution of 1:40
				Case control Comparator Population (309)	Matched	Matched	*	No clinical criteria applied	CCP2, IgM-RF	As above

* Data not reported.

ACPA, anticitrullinated protein autoantibodies; ACR, American College Rheumatology; CarP, anticarbamylated protein antibodies; CCP2, anticyclic citrullinated antibodies 2; DODSR and WRMC Military, Department of Defence Serum Repository and Walter Reed Medical Centre; EPIC, European Prospective Investigation into Cancer and Nutrition; EULAR, European League Against Rheumatism; IgA-RF, immunoglobulin A rheumatoid factor; IgG-RF, immunoglobulin G rheumatoid factor; IgM-RF, immunoglobulin M rheumatoid factor; RA, rheumatoid arthritis; ULN, upper limit of normal.

**Table 2:** Prospective cohort studies and randomised control trials

Source region (country)	Author	Data provided by authors	Study cohort	Type of study	Number of individuals	Sex (% female)	Intervention	Age (mean) years	Ever smokers (%)	Cohort entry selection criteria yes/no (% if reported)			Outcome	Autoantibody assessed	Threshold for positivity
										FDR	Arthralgia	Imaging			
**Region of the Americas**															
USA	Bemis et al^53^	No	SERA	Prospective Observational	71	77.9	No	48	*	Yes (100)	No (63.6)	No	≥1 swollen joint	CCP2, IgM-RF, CCP3	≥1 ULN
Canada	Tanner *et al*^31^	Yes	North American Indigenous	Prospective Observational	85	*	No	*	*	Yes (75)	No (56)	No	≥1 swollen joint	CCP3	≥1 ULN
**European Region**															
UK	Duquenne *et al*^30^	No	Leeds	Prospective Observational	455	73	No	50	53	No	Yes	No	≥1 swollen joint	CCP2, IgM-RF	≥1 ULN
	Pratt *et al*^8^	Yes	Newcastle	Prospective Observational	41	68	No	47	*	No	Yes (100)	No	≥1 swollen joint	CCP2	≥1 ULN
Netherlands	Ten Brinck et al^9^	No	Leiden Clinically Suspicious Arthralgia Cohort (2012–2015)	Prospective Observational	32	78	No	44	22	No (30)	Yes (100)	No	≥1 swollen joint	CCP2, IgM-RF	≥1 ULN
	Bos *et al*^11 54^	No	Amsterdam Reade (2004–2007)	Prospective Observational and dexamethasone RCT - placebo arm	54 and 41	77 and 66	No + dexamethasone	49 and 48	*	No	Yes (100)	No	≥1 swollen joint or 1987 criteria	CCP2, IgM-RF	≥1 ULN
	Van Beers-Tas *et al*^10^	Yes	Amsterdam Reade (2009–2015)	Prospective Observational	91	*	No	*	*	No	Yes (100)	No	≥1 swollen joint	CCP2, IgM-RF	≥1 ULN
Sweden	Eloff *et al*^55^	Yes	TIRx, Linkoping	Prospective Observational	82	81	No	52	48	No	Yes (100)	No	≥1 swollen joint	CCP2, IgM-RF	≥1 ULN, 30 U/mL
	Erlandsson *et al*^56^	Yes	Gothenburg and Umea	Prospective Observational	14	*	No	*	*	No	Yes (100)	No	RA 2010 ACR/EULAR criteria	CCP2, RF	≥1 ULN, 20 U/mL or 95% specificity in controls
Switzerland	Gilbert *et al*^57^	Yes	SCREEN-RA	Prospective Observational	80	*	No	*	*	Yes (100)	No (0%)†	No	>1 swollen joint	CCP2 or CC3	≥1 ULN
**Multi-Region**	Van Boheemen *et al*^12^	No	STAP-RA	RCT – placebo arm	31	81	Atorvastatin	46	42	No	Yes (100)	No	≥1 swollen joint or 2010 ACR/EULAR criteria.	CCP2	ACPA) ≥3 x ULN or both ACPA (≥7×1 ULN) and rheumatoid factor (RF; ≥1 ULN),
	Cope *et al*^17^	Yes	APIPPRA	RCT – placebo arm	103	79	Abatacept	49	79	No	Yes (100)	No	≥3 swollen joints or 2010 ACR/EULAR criteria (100% concordance)	CCP2, IgM-RF	ACPA) ≥3 x ULN or both ACPA (≥1 ULN) & (RF (≥1 ULN),

* Data not reported.

† Defined as >4 of EULAR clinically suspect arthralgia criteria.^18^

ACPA, anticitrullinated protein autoantibodies; ACR, American College Rheumatology; APIPPRA, arthritis prevention in the pre-clinical phase of rheumatoid arthritis with abatacept; CarP, anticarbamylated protein antibodies; CCP2, anti cycliccitrullinated antibodies 2; CCP3, anticyclic citrullinated antibodies 3; EULAR, European League Against Rheumatism; FDR, first degree relative with RA; IgM-RF, immunoglobulin M rheumatoid factor; RA, rheumatoid arthritis; RCT, randomised controlled trial; SCREEN-RA, Evaluation of a SCREENing strategy for Rheumatoid Arthritis; SERA, Studies of the Aetiology of Rheumatoid Arthritis; STAP-RA, STAtins to Prevent Rheumatoid Arthritis; TIRx, X-tra timely rheumatology follow-up; ULN, upper limit of normal.

To calculate the relative risk of RA, we stratified studies according to autoantibody examined.

**Figure 2:**
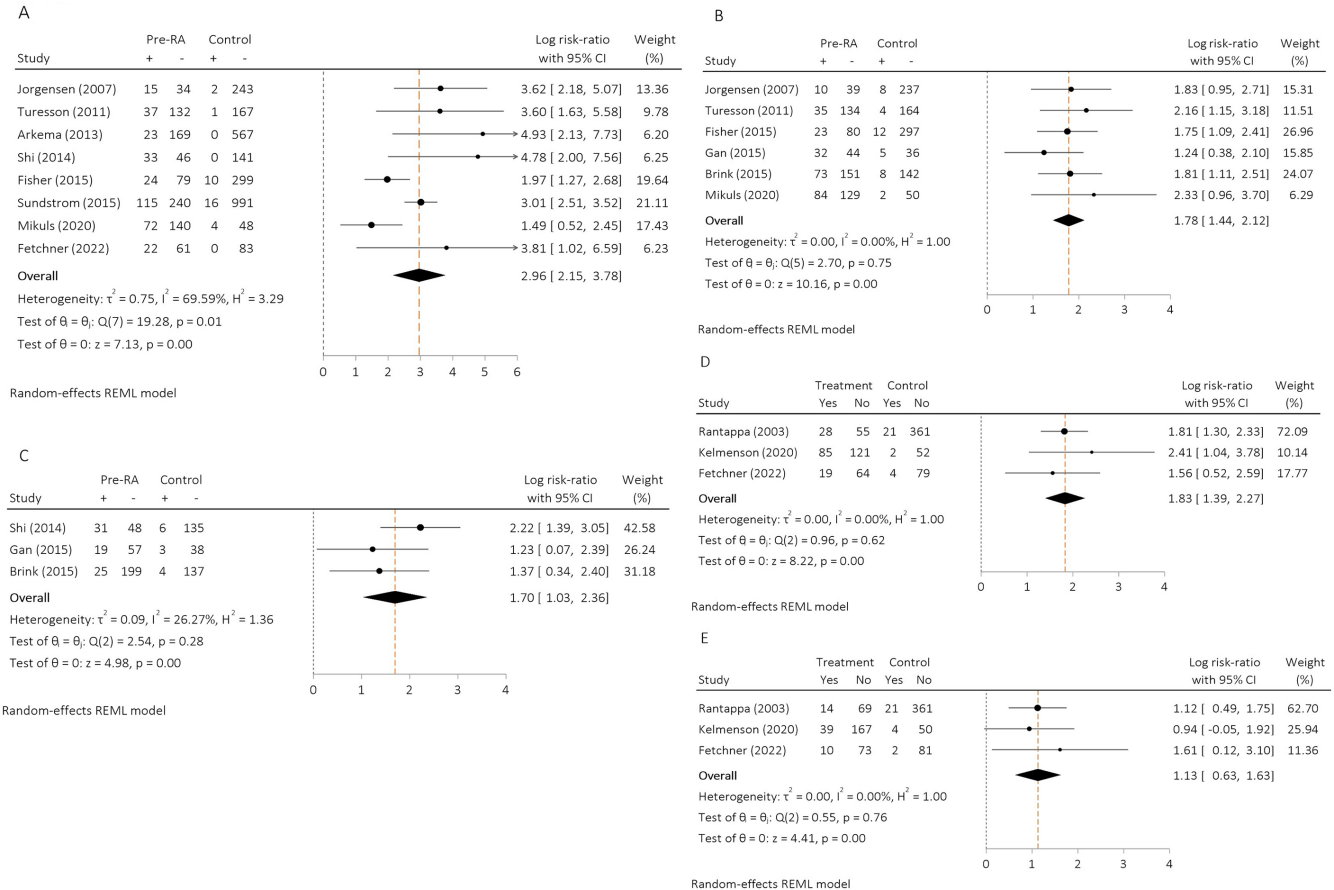
Pooled log risk ratio of developing rheumatoid arthritis (RA) in individuals positive for (**A**) anti-citrullinated protein autoantibodies (ACPA); (**B**) immunoglobulin M rheumatoid factor (IgM-RF); (**C**) anticarbamylated autoantibodies (anti-CarP); (**D**) immunoglobulin A rheumatoid factor (IgA-RF); (**E**) immunoglobulin G rheumatoid factor (IgG-RF) compared with autoantibody negative individuals. REML, restricted maximum likelihood.

The relative risk of RA in CCP2 positive individuals was calculated using seven cohorts from the USA, Sweden, Norway and a pan-European cohort (figure 2A). A total of 1242 patients with RA and 2572 matched controls were analysed with pre-RA serum samples tested between 7.5 years and 9.5 years before diagnosis. The pooled relative risk of developing RA was 19.3 times higher in CCP2 positive individuals compared with CCP2 negative individuals (log risk-ratio 2.96; 95% CI 2.15 to 3.78). However, Egger’s test indicated potential small sample effects (p=0.014), confirmed by visual inspection of the funnel plot (online supplemental figure 1). A PEESE bias-adjusted estimate of the pooled relative risk was calculated and estimated that the risk of developing RA was 11.3 times higher in CCP2 positive individuals (log risk-ratio 2.43) and remained statistically significant (p=0.002).

The relative risk of RA in IgM-RF positive individuals was calculated using six cohorts from the USA, Sweden, Norway, the Netherlands and a pan-European cohort (figure 2B). A total of 834 patients with RA and 965 matched controls were included, with pre-RA serum samples tested between 5 years and 10 years before diagnosis. The pooled relative risk ratio of developing RA was 5.9 times higher in IgM-RF positive individuals compared with IgM-RF negative individuals (log risk ratio 1.78; 95% CI 1.44 to 2.12). However, Egger’s test indicated potential small sample effects (p=0.011; confirmed by visual inspection of the funnel plot (online supplemental figure 1). A PEESE bias-adjusted estimate of the pooled relative risk was calculated and estimated that the risk of developing RA was 4.5 times higher in IgM-RF positive individuals compared with controls (log risk-ratio 1.50) and remained statistically significant (p<0.01).

The relative risk of RA in anti-CarP positive individuals was calculated using three cohorts from the USA, the Netherlands and Sweden (figure 2C). A total of 379 patients with RA and 323 matched controls were analysed with pre-RA serum samples tested between 1.4 years and 10 years before diagnosis. The pooled relative risk of developing RA was 5.5 times higher in anti-CarP positive individuals compared with anti-CarP negative individuals (log risk-ratio 1.70; 95% CI 1.03 to 2.36). Egger’s test did not indicate small sample effects (p=0.061).

The relative risk of RA in IgA-RF and IgG-RF positive individuals was calculated using two cohorts from the USA and Sweden (figure 2D,E). A total of 372 patients with RA and 519 matched controls were included, with pre-RA samples tested 2.5–8.4 years before diagnosis. The pooled relative risk of developing RA in IgA-RF positive individuals was 6.2 times higher (log risk-ratio 1.83; 95% CI 1.39 to 2.27) and 3.1 times higher (log risk-ratio 1.13; 95% CI 0.63 to 1.63) in IgG-RF positive individuals respectively, compared with IgA-RF and IgG-RF negative individuals. Egger’s test did not indicate small sample effects for IgA-RF (p=0.135) or IgG-RF (p=0.251) analysis.

The leave-one-out meta-analysis for all autoantibodies revealed no study was deemed influential and the results of this sensitivity analysis are presented in online supplemental figure 1.

To estimate the cumulative incidence of RA in autoantibody positive individuals, we reconstructed patient level data from KM survival curves to generate a KM best fit survival model. Pooling of data was according to autoantibody, presence of joint symptoms, FH of RA and imaging changes (characteristics which were most often used to enrol cohorts in the included studies; see online supplemental table 4). The most common autoantibody used for enrolment into studies was anti-CCP, using commercial assays anti-CCP2 and anti-CCP3.

**Figure 3:**
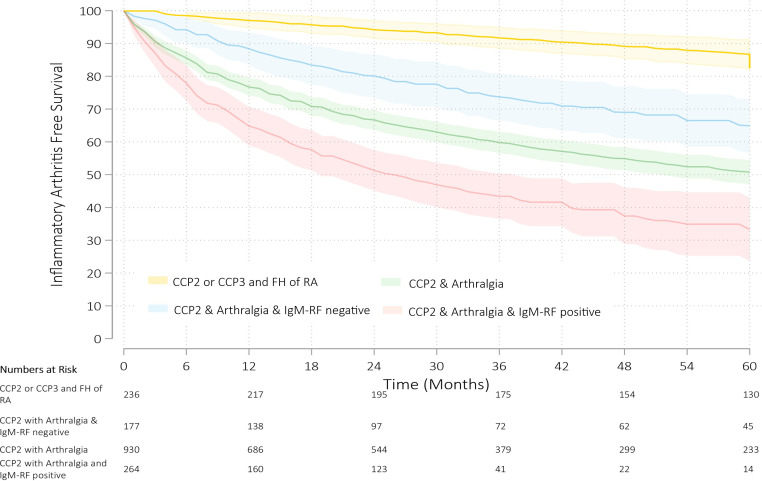
Predicted inflammatory arthritis free survival model in CCP2 or CCP3 positive individuals with a first or second degree relative with RA (yellow); CCP2 individuals with arthralgia (green); CCP2 positive with arthralgia and IgM-RF negative (blue); CCP2 positive with arthralgia and IgM rheumatoid factor positive (red), calculated using pooled mixed effects model. Shaded areas represent 95% CI. CCP2, anticyclic citrullinated antibodies 2; CCP3, anticyclic citrullinated antibodies 3; FH, family history, IgM-RF, immunoglobulin M rheumatoid factor; RA, rheumatoid arthritis.

Studies were pooled, and survival times were described across four cohorts of individuals: (1) CCP2 positive with arthralgia (n=930); (2) CCP2 and IgM-RF positive with arthralgia (n=264); (3) CCP2 positive and IgM-RF negative with arthralgia (n=177); (4) CCP2 or CCP3 positive and first or second degree relative with RA (referred to as FH of RA) (n=236). While two eligible studies incorporated MRI and three incorporated ultrasonography, the imaging protocols were too heterogenous to draw robust conclusions.^13 16 17 25 26^ The highest predicted cumulative incidence of inflammatory arthritis over any period of observation was in CCP2 and IgM-RF positive individuals with arthralgia, with an estimated cumulative incidence of 67% (95% CI 57% to 76%) at 60 months. The lowest predicted cumulative incidence of RA was in the cohort of CCP2 or CCP3 positive individuals with a FH of RA (with or without arthralgia) at 13% (95% CI 9% to 18%) at 60 months. Cumulative incidence at 6 months, 12 months, 24 months, 36 months, 48 months and 60 months for all cohorts is presented in table 3. The pooled KM survival model with 95% CIs for the four cohorts over 60 months is presented in figure 3.

**Table 3:** Cumulative incidence of inflammatory arthritis in 4 cohorts of CCP2 positive individuals

Cohorts	Number of studies (total number of study subjects)	Cumulative incidence of inflammatory arthritis % at time in months (95% CI)					
		6	12	24	36	48	60
CCP2 or CCP3 positive and first or second degree relative with RA	3 (236)	1.5 (0.6 to 2.3)	3.0 (0.6 to 5.4)	5.8 (2.9 to 8.6)	8.3 (5.0 to 11.6)	10.9 (7.1 to 14.7)	13.3 (8.6 to 18.0)
CCP2 positive and arthralgia and IgM-RF negative	5 (177)	5.8 (1.9 to 9.8)	11.6 (6.8 to 16.4)	19.9 (13.3 to 26.6)	26.3 (19.1 to 33.6)	31.0 (23.0 to 39.0)	35.1 (26.8 to 43.4)
CCP2 positive and arthralgia	8 (930)	14.4 (12.1 to 16.6)	23.3 (20.6 to 25.9)	33.3 (30.3 to 36.3)	40.2 (37.0 to 43.5)	45.1 (41.6 to 48.6)	49.2 (45.5 to 52.9)
CCP2 positive and arthralgia and IgM-RF positive	7 (264)	22.1 (16.7 to 27.4)	35.2 (29.3 to 41.2)	48.7 (42.5 to 54.9)	56.6 (49.7 to 63.5)	62.6 (54.0 to 71.2)	66.7 (57.1 to 76.4)

CCP2, anticyclic citrullinated antibodies 2; CCP3, anticyclic citrullinated antibodies 3; IgM-RF, immunoglobulin M rheumatoid factor; RA, rheumatoid arthritis.

Substratification of CCP2 positive individuals with arthralgia using IgM-RF autoantibodies produced a marked divergence in the predicted rate of progression, apparent as early as 6 months and persisting out to 60 months. At 6 months, the predicted cumulative incidence of inflammatory arthritis in IgM-RF positive individuals was 22% (95% CI 17% to 27%) compared with 6% (95% CI 2% to 10%) of IgM-RF negative individuals; at 60 months this had increased to 67% (95% CI 57% to 76%) and 35% (95% CI 27% to 43%), respectively.

We also observed a change in the risk of progression over time for the three cohorts who had symptoms of arthralgia. There was a similar higher event rate in the earlier periods of follow-up which decreased over time, following a pattern of exponential decay. The highest risk period for developing inflammatory arthritis was during the first 24 months across all these groups, reflected in the change in the hazard function across time (online supplemental figure 5). The initial 24-month period was defined as the interval following first presentation to a rheumatologist. In contrast, the cohort with CCP2 or CCP3 and FH of RA appeared to have a constant event rate over time, with the risk of progression at later time periods similar to the initial period of observation even though these studies had longer periods of follow-up (up to 144 months).

The individually reconstructed KM survival curves with the pooled model are presented in online supplemental figure 3 to allow for a visual comparison of the pooled studies. The overall KM survival curves with the pooled model are presented in online supplemental figure 4 to allow visual comparison of the predicted model and reported event rates.

## Discussion

In the largest systematic review to date, we have examined the relationship between autoantibodies and risk of progression to RA. In the first part of our analysis, we found that the presence of autoantibodies confers between 3.1 times to 19.3 times higher risk of developing RA, as compared with autoantibody negative individuals. The highest risk is conferred by ACPA, which is in line with a previous meta-analysis, indicating that, of the individual autoantibodies, ACPA confers the highest OR for developing RA.^27^ The range of time over which pre-RA samples underwent testing varied widely and although we could not account for this in the analyses, separate studies of serial samples across timepoints have shown that the proportion of individuals positive for an autoantibody increases and is highest at the time nearest to the onset of RA.^28^ It is important to note that the retrospective case-control studies, while useful for identifying associations, did not allow for direct estimation of absolute risk.

In contrast, the second part of our analysis, which used prospective cohort data, allowed for the estimation of risk over time, providing a more robust assessment of incidence and temporal relationships. In this analysis, the fastest rate of progression and the highest overall cumulative incidence appears to be associated with positivity for both CCP2 and IgM-RF in combination with arthralgia, as compared with individuals who are CCP2 positive alone. A recent 5-year follow-up of 641 patients with arthralgia screened for ACPA or RF also found that the highest rates of progression were for ACPA and IgM-RF positive patients.^29^

Crucially for patients and clinicians, the risk of progression changes over time: when arthralgia exists, in the presence of both CCP2 and IgM-RF, approximately 50% of the entire cohort were predicted to progress within the first 24 months of presentation to rheumatologists; after this time point the rate of progression reduced markedly, with only a further 14% progressing over the next 24 months. When focusing on only those individuals who developed RA over the 60 months of follow-up, approximately 50% were predicted to do so within the first 12 months. Interestingly, despite cross-country differences in access to specialist rheumatology services, the pattern was consistently observed (see online supplemental figure 3B). This change in risk over time has been shown in studies with follow-up to 10 years, where the longer an individual remains arthritis-free, the less likely they are to progress to RA.^30^

The reasons for this change in risk over time have yet to be fully elucidated, though current research points to several possibilities.

The autoantibody profiles of at-risk individuals appear to change over time, including a possible conversion to a seronegative state^31^ and have been shown to influence rates of progression to RA. The phenomenon of epitope spreading,^32^ isotype switching,^33^ as well as the kinetics of changes in autoantibody levels^28^ and the number of autoantibodies^17^ have all been shown to predict progression to RA. Interestingly, IgG-ACPA Fab V-domain glycosylation in at-risk individuals was increased^34 35^ in those who progressed to RA compared with those who did not. A third possible mechanism is the expansion of T helper immune cell subsets^36^ or dysregulation of B and T cell pools in at-risk individuals who progressed.^37^ Future studies will need to evaluate if these biologic processes differ between at-risk individuals who develop RA in (a) short timeframe (eg, 12–24 months); (b) longer time frame (24–60 months) or (c) or not at all. It is also possible that these immunologic risk factors interact with other well-known, potentially reversible environmental risk factors (eg, smoking) or lifestyle behaviours, which further modulates their risk over time. In contrast, individuals with a FH of RA had more constant event rates over time, and it is not fully understood why this may differ. It may be that the approach for identifying at-risk individuals with joint symptoms biases towards a subset who will rapidly develop RA, and individuals with a FH of RA are identified at an earlier stage of RA development.

In our study, the presence of two autoantibodies appeared to confer a greater risk of progression to RA. However, in clinical practice, the ability to investigate multiple autoantibodies may be limited by cost, feasibility or local access to relevant assays, and many of these individuals defined as ‘CCP2 positive’ are likely to be positive for other autoantibodies. The CCP2 and IgM RF positive/negative individuals are therefore likely to represent *subsets* of the CCP2 positive population rather than a separate cohort. In keeping with this, when these at-risk individuals have been examined for additional autoantibodies, such as anti-CarP or IgA-ACPA, different combinations of autoantibody positivity have emerged,^9 17^ which have been shown to further influence progression rates to RA.

Testing for multiple autoantibodies and using this to stratify individuals has been used in other autoimmune diseases. In type 1 diabetes (an autoimmune condition with predictive autoantibodies), prospective longitudinal studies were used to define distinct identifiable stages prior to the onset of symptoms; stage 1 was defined by the presence of two or more autoantibodies, stage 2 as the presence of dysglycaemia and stage 3 as the onset of clinical disease.^38^ In our analysis, we draw a similar parallel with the presence of two or more autoantibodies conferring a much greater predictive value for disease onset than one autoantibody alone and the presence of joint symptoms conferring a greater risk of progression compared with those individuals who were asymptomatic.

For clinicians, especially those working within constrained public healthcare systems, assessment of these factors may help to both stratify individuals at risk and judge the time for which these patients should be monitored most closely. For individuals, it may also help to understand their individual risk of progression, which has been shown to be a key facet of decision making for their willingness to engage with preventative treatments in the research setting.^39^ Importantly, our study has shown that not all individuals positive for autoantibodies, even with joint complaints, appear to develop RA within the timeframe of these studies, some of which extend up to 5 years.

A strength of our work lies in the application of an innovative digital software tool and statistical programme to extract and generate patient level data. This allowed for the synthesis of a large dataset of individuals at risk, without the need for transfer of patient data, and we were able to report progression rates up to 5 years. The willingness of authors to contribute and clarify data greatly improved our ability to include more studies as well as the recent publication of several large RCTs, allowing us to overcome previous difficulties in being able to meta-analyse these cohorts.

Substantial challenges in confirming and aggregating data for the cohort studies exist, although many were at low risk of bias. Review of the literature revealed that several cohorts have been published assessing different autoantibodies, at different timepoints, with varied levels of primary data reporting. Many RCTs and prospective cohorts also applied additional clinical, history and imaging requirements, leading to difficulties in the pooling of cohorts. Again, after direct communication with authors and principal investigators, we were able to obtain further data for many of the subpopulations we studied and avoided duplication in pooled data. As we had only the time to event data for individuals, we were unable to examine and stratify for other risk factors for progression such as the duration of arthralgia or smoking history which have been shown to be important. We have also not examined the immunological mechanisms underlying the increased risk associated with autoantibody positivity, as this was not the focus of our study.

Limitations of our study include the definition of joint-related symptoms, which for the purposes of our analysis were defined as any musculoskeletal symptom. In the primary care setting, however, musculoskeletal complaints are very common, and recent work has tried to identify specific joint symptoms that can discriminate between inflammatory and non-inflammatory arthralgia. Most of the eligible studies in our analysis were initiated prior to the 2017 EULAR definition of ‘clinically suspect arthralgia’. Therefore, our results may represent a lower progression rate for joint-related symptoms, and we would agree with a recent systematic literature review and risk stratification which encouraged use of this tool for future research in the field to help improve risk stratification and allow homogenisation of the cohorts.^19 20^

Another limitation was the outcome of studies; for the purposes of our analysis, this was defined as the development of ≥1 joint with clinical arthritis or meeting RA 2010 ACR/EULAR or 1987 ACR criteria. It has been noted that only having a single swollen joint may lead to the diagnosis of undifferentiated arthritis (UA) rather than RA. However, in the context of positive autoantibodies, a recent systematic literature review noted that studies assessing patients classified as UA were based on failure to meet ‘now outdated RA classification criteria’ and it is therefore likely that a significant proportion of these patients in these studies with UA would classify as RA based on contemporary criteria.^40^ In our analysis, when RCTs^12^ and observation cohorts^30^ assessed both outcomes (the development of ≥1 swollen joint or 2010 RA/EULAR criteria), there was a strong correlation (≥90% concordance).

Importantly, there are no diagnostic criteria for RA and the clinical relevance of a swollen joint in this context is likely to be important—in the TREAT-EARLIER trial which required two swollen joints persisting for >2 weeks or ≥1 joint with clinical arthritis meeting the 2010 ACR/EULAR criteria, participants who developed clinical arthritis but did not meet the 2010 ACR/EULAR RA classification criteria were all clinically diagnosed with RA.^13^

Other important limitations were present, particularly in the first part of the analysis examining retrospective cohorts. We were unable to control for confounding variables due to a lack of reported data. Additionally, the small number of studies and sample sizes for certain autoantibodies introduced sparse data bias, potentially affecting the precision of effect estimates. This also limited our ability to reliably assess between-study heterogeneity.

Importantly, there are additional factors to consider when using autoantibodies as predictors for RA, which we were unable to examine in our work. The levels of autoantibodies are a key feature which has been shown to further stratify progression rates within at-risk individuals.^9 41^ The StopRA trial, which recruited individuals using CCP3 ≥40, reported much higher rates of progression than our CCP2 or CCP3 positive cohort (32.9% in the placebo arm at 36 months).^15^ The recent EULAR/ACR risk stratification tool published in May 2025 examined 2293 individuals at risk of RA, of whom 48% were ACPA positive.^20^ In a simplified risk model using six clinical and serological variables, ‘high titre’ ACPA and RF (defined as≥3 the upper limit of normal) were given considerable weighting, contributing 8 and 4 points respectively out of a maximum 26 points. Since the discovery of ACPA and the first generation anti-CCP test becoming commercially available, ACPA assays continue to be developed in the commercial and research settings (eg, CCP2, CCP3, CCP3.1, anti-mutated citrullinated vimentin). Each test may perform differently when estimating the risk of future RA^42^ and further work is required to determine how these should be used in pre-RA. As the number of studies addressing this question was limited, we were unable to compare and draw firm conclusions on the predictive value of different assays.

A key consideration in the selection of any variable used for prediction is how these may vary over time and the utility of repeated measurements. In our study, a single autoantibody measurement at baseline was used to stratify individuals. However, retrospective studies examining serial measurements over time in autoantibody positive at-risk cohorts have shown levels rise significantly in the years preceding onset.^28 32 43^ Conversely, a recent ultrasound study examining at-risk CCP positive individuals found the subclinical imaging changes resolved in approximately half of all individuals within 12 months.^44^ How changes in risk factors over time impact progression rates to RA has yet to be fully understood and warrants further investigation.

As we enter the era of prevention of clinical disease, with two recent RCTs reporting successful delay of the onset of RA, the need for a standardised approach to identifying individuals at highest risk is becoming more urgent. Despite the inherent challenges of prediction and the clinical heterogeneity of RA, clear patterns of progression are emerging of use to clinicians, patients and researchers alike. We can now provide more precise risk estimates, changing across time, for autoantibody positive individuals at risk of RA.

## Supplementary material

10.1136/rmdopen-2025-006368 online supplemental file 1

## Data Availability

Data are available upon reasonable request.
