# Supplementary material for: Autoantibodies as predictors of progression to rheumatoid arthritis: a systematic review and meta-analysis
Source: RMD Open. 2026 Feb 11;12(1):e006368. doi: 10.1136/rmdopen-2025-006368 (PMC12911842; doi:10.1136/rmdopen-2025-006368)
Supplement: online supplemental file 1 [file rmdopen-12-1-s001.docx]

Supplementary data

1. Search Strategy…………………………………………………………………………………....3
2. Bias Assessments………………………………………………………………………...………..5

- **Supplementary Table 1**. Randomised Control Trials bias assessment using Risk of Bias 2 tool) ……………………………………………………………………………………..........5
- **Supplementary Table 2.** Case Control Studies bias assessment using Newcastle – Ottawa Quality Assessment tool (NOS)………………………………………………..……………..5
- **Supplementary Table 3.** Prospective Cohort Studies bias assessment using Newcastle – Ottawa Quality Assessment tool (NOS)………………………………………………..…..…6
- **Supplementary Figure 1.** Leave one out meta-analysis and funnel plots for (A) CCP2 (B) IgM-RF (C) anti-CarP (D) IgA-RF and (E) IgG-RF………………………………………. 7-8

1. Pooling of Eligible Studies ……………………………………………………………………….9

- **Supplementary Table 4**. Eligible studies of ACPA positive patients pooled according to other factors………………………………………………………………………………..9-10
- **Supplementary Figure 2** Individual Kaplan Maeir Survival Curves of Inflammatory Arthritis Free Survival in ACPA positive cohorts & pooled survival models…...……… 10-11
- **Supplementary Figure 3**. Overall Kaplan Maeir Survival Curves of Inflammatory Arthritis Free Survival in ACPA positive cohorts & pooled survival models…….……….…………..12
- **Supplementary Figure 4.** Hazard function for CCP (anti-citrullinated autoantibodies) positive individuals in combination with other factors. …………………………………….13

1. Supplementary Results – Imaging Studies…………………………………………….....………14

- **Supplementary Table 5**. Eligible studies of ACPA positive patients pooled according to imaging factors……………………………………………………………..………………..15

1. Supplementary Results – Ultrasound…………………………………………….....……………17

- **Supplementary Figure 5**: KM survival curves for CCP2 positive individuals with Arthralgia and power doppler changes on Ultrasound………………………………………………..….17

1. Supplementary Results – MRI…………………………………………………………………...18

- **Supplementary Figure 6:** KM survival curves for CCP2 positive individuals with Arthralgia who have MRI changes of osteitis, tenosynovitis, or synovitis………………………………18

1. **Search Strategy**

The below search strategy was used to search the OVID database. To allow for de-duplication of data, the search was conducted in parts from inception to 2025 (line 29) as automated de-duplication in OVID is limited to 6000 studies. Search operators with ‘*’ allowed for the inclusion of term variation.

| Set | Search Statement |
| --- | --- |
| 1. | autoantibod*.mp. [mp=ti, ab, hw, tn, ot, dm, mf, dv, kf, fx, dq, bt, nm, ox, px, rx, ui, sy, cw] |
| 2. | antibod*.mp. [mp=ti, ab, hw, tn, ot, dm, mf, dv, kf, fx, dq, bt, nm, ox, px, rx, ui, sy, cw] |
| 3. | AMPA.mp. [mp=ti, ab, hw, tn, ot, dm, mf, dv, kf, fx, dq, bt, nm, ox, px, rx, ui, sy, cw] |
| 4. | citrullinated.mp. [mp=ti, ab, hw, tn, ot, dm, mf, dv, kf, fx, dq, bt, nm, ox, px, rx, ui, sy, cw] |
| 5. | CarP.mp. [mp=ti, ab, hw, tn, ot, dm, mf, dv, kf, fx, dq, bt, nm, ox, px, rx, ui, sy, cw] |
| 6. | CCP.mp. [mp=ti, ab, hw, tn, ot, dm, mf, dv, kf, fx, dq, bt, nm, ox, px, rx, ui, sy, cw] |
| 7. | ACPA.mp. [mp=ti, ab, hw, tn, ot, dm, mf, dv, kf, fx, dq, bt, nm, ox, px, rx, ui, sy, cw] |
| 8. | RF.mp. [mp=ti, ab, hw, tn, ot, dm, mf, dv, kf, fx, dq, bt, nm, ox, px, rx, ui, sy, cw] |
| 9. | Rheumatoid Factor.mp. [mp=ti, ab, hw, tn, ot, dm, mf, dv, kf, fx, dq, bt, nm, ox, px, rx, ui, sy, cw] |
| 10. | Ab.mp. [mp=ti, ab, hw, tn, ot, dm, mf, dv, kf, fx, dq, bt, nm, ox, px, rx, ui, sy, cw] |
| 11. | pre.mp. [mp=ti, ab, hw, tn, ot, dm, mf, dv, kf, fx, dq, bt, nm, ox, px, rx, ui, sy, cw] |
| 12. | prior.mp. [mp=ti, ab, hw, tn, ot, dm, mf, dv, kf, fx, dq, bt, nm, ox, px, rx, ui, sy, cw] |
| 13. | before.mp. [mp=ti, ab, hw, tn, ot, dm, mf, dv, kf, fx, dq, bt, nm, ox, px, rx, ui, sy, cw] |
| 14. | at risk.mp. [mp=ti, ab, hw, tn, ot, dm, mf, dv, kf, fx, dq, bt, nm, ox, px, rx, ui, sy, cw] |
| 15. | pre-clinical.mp. [mp=ti, ab, hw, tn, ot, dm, mf, dv, kf, fx, dq, bt, nm, ox, px, rx, ui, sy, cw] |
| 16. | rheumatoid arthritis.mp. [mp=ti, ab, hw, tn, ot, dm, mf, dv, kf, fx, dq, bt, nm, ox, px, rx, ui, sy, cw] |
| 17. | arthralgia.mp. [mp=ti, ab, hw, tn, ot, dm, mf, dv, kf, fx, dq, bt, nm, ox, px, rx, ui, sy, cw] |
| 18. | sub-clinical.mp. [mp=ti, ab, hw, tn, ot, dm, mf, dv, kf, fx, dq, bt, nm, ox, px, rx, ui, sy, cw] |
| 19. | MAA.mp. [mp=ti, ab, hw, tn, ot, dm, mf, dv, kf, fx, dq, bt, nm, ox, px, rx, ui, sy, cw] |
| 20. | 1 or 2 or 3 or 4 or 5 or 6 or 7 or 8 or 9 or 10 or 19 |
| 21. | 11 or 12 or 13 or 14 or 15 or 18 |
| 22. | 16 or 17 |
| 23. | 20 and 21 and 22 |
| 24. | limit 23 to ("adult (19 to 44 years)" or "young adult and adult (19-24 and 19-44)" or "middle age (45 to 64 years)" or "middle aged (45 plus years)" or "all aged (65 and over)" or "aged (80 and over)") |
| 25. | limit 24 to english language |
| 26. | limit 25 to human |
| 27. | limit 26 to humans |
| 28. | limit 27 to (adult <18 to 64 years> or aged <65+ years>) |
| 29. | limit 28 to yr="1960 -2010" |
| 30. | Remove duplicates from 29 |

The below search strategy was used to search the SCOPUS database. De-duplication was performed by unticking studies labelled as editorial, book chapters or retracted.

 ( TITLE-ABS-KEY ( progression OR leading OR development OR onset ) ) AND ( TITLE-ABS-KEY ( autoantibod* OR antibod* OR rf OR "rheumatoid factor" OR ccp OR acpa OR citrullinated OR ampa OR ab OR carp ) ) AND ( TITLE-ABS-KEY ( pre OR prior OR before OR at-risk OR pre-clinical ) ) AND ( TITLE-ABS-KEY ( "rheumatoid arthritis" OR "clinically suspicious arthralgia" OR arthralgia ) ) ) AND ( LIMIT-TO ( DOCTYPE , "ar" ) OR LIMIT-TO ( DOCTYPE , "re" ) OR LIMIT-TO ( DOCTYPE , "cp" ) OR LIMIT-TO ( DOCTYPE , "Undefined" ) ) AND ( LIMIT-TO ( LANGUAGE , "English" ) ) AND ( LIMIT-TO ( EXACTKEYWORD , "Human" ) OR LIMIT-TO ( EXACTKEYWORD , "Humans" ) )

**Supplementary table 1. Randomised controlled trials bias assessment using (Risk of bias 2 (RoB2) tool)**

| Study | Random sequence generation | Allocation concealment | Blinding patients and personnel | Blinding outcome assessment | Incomplete outcome data | Selective reporting | Overall |
| --- | --- | --- | --- | --- | --- | --- | --- |
| Bos et al. 2010 ^1^ |  |  |  |  |  |  |  |
| Van Boheemen et al. 2021^2^ |  |  |  |  |  |  |  |
| Krijbolder et al. 2022^3^ |  |  |  |  |  |  |  |
| Cope et al. 2024^4^ |  |  |  |  |  |  |  |
| Rech et al 2024^5^ |  |  |  |  |  |  |  |

Bias assessed using Cochrane risk of bias tool. Green = low risk of bias, Red = high risk of bias, Yellow = unknown risk of bias.

**Supplementary table 2. Case Control Studies bias assessment using Newcastle – Ottawa Quality Assessment tool (NOS)**

| **Study** | **Selection** | | | | **Comparability** | | **Exposure** | | | |
| --- | --- | --- | --- | --- | --- | --- | --- | --- | --- | --- |
|  | Case Definition | Representativeness | Selection of Controls | Definition of Controls | Study controls for Age | Study controls for sex smoking location | Ascertainment of Exposure | Same method of ascertainment | Non-response rate | |
| Rantapaa et al. 2003^6^ | * | * | * | * | * | * | * | * | * | |
| Jorgensen et al. 2007^7^ |  | * | * | * | * |  | * | * |  | |
| Turesson et al. 2010^8^ | * | * | * | * | * | * | * | * | * | |
| Shi et al. 2013^9^ | * | * | * |  | * | * | * | * |  | |
| Arkema et al. 2013^10^ | * | * | * | * | * | * | * | * |  | |
| Gan et al. 2015^11^ | * | * | * | * | * | * | * | * |  | |
| Brink et al. 2015^12^ | * | * | * | * | * | * | * | * | * | |
| Sundstrom et al. 2015^13^ | * | * | * |  | * | * | * | * | * | |
| Fisher et al. 2015^14^ | * | * | * |  | * | * | * | * |  | |
| Kelmenson et al. 2020^15^ | * | * | * | * | * | * | * | * |  |  |
| Mikuls et al. 2020^16^ | * | * | * | * | * | * | * | * |  | |
| Fetchner et al. 2022 | * | * | * | * | * | * | * | * |  | |

Green = low risk of bias, Red = high risk of bias, Yellow = unknown risk of bias.

**Supplementary table 3. Prospective Cohort Studies bias assessment using Newcastle – Ottawa Quality Assessment tool (NOS)**

| **Study** | **Selection** | | | | **Comparability** | | **Exposure** | | |
| --- | --- | --- | --- | --- | --- | --- | --- | --- | --- |
|  | Representativeness | Selection of Non exposed Cohort | Ascertainment of Exposure | Demonstration that outcome of interest not present at start of study | Study controls for Age | Study controls for sex smoking location | Ascertainment of Outcome | Follow up long enough | Adequacy of follow up of cohorts |
| Bemis et al. 2020^17^ | * | N/A | * | * | N/A | N/A | * | * | * |
| Duquenne et al. 2024^18^ | * | N/A | * | * | N/A | N/A | * | * | * |
| Pratt et al. (unpublished) | -- |  | -- | -- |  |  | -- | -- | -- |
| Ten Brinck et al. 2017^19^ | * | N/A | * | * | N/A | N/A | * | * | * |
| Bos et al. 2010^20^ | * | N/A | * | * | N/A | N/A | * | * | * |
| Van Beers-Tas et al. 2018^21^ |  | N/A | * | * | N/A | N/A | * | * | * |
| Eloff et al. 2020^22^ | * | N/A | * | * | N/A | N/A | * | * | * |
| Erlandsson et al. 2018^23^ | * | N/A | * | * | N/A | N/A | * | * | * |
| Gilbert et al. 2021^24^ | * | N/A | * | * | N/A | N/A | * | * | * |
| Tanner et al 2019^25^ | * | N/A | * | * | N/A | N/A | * | * | * |

Green = low risk of bias, Red = high risk of bias, Yellow = unknown risk of bias.

**C**

**B**

**A**

**D**

**E**

**Supplementary Figure 1. Leave one out met-analysis and funnel plots for A) CCP2 B) IgM-RF C) anti-CarP D) IgA-RF and E) IgG-RF**

**Supplementary Table 4. Eligible studies of ACPA positive patients pooled according to other factors: Arthralgia; Arthralgia & IgM-RF positive; Arthralgia & IgM-RF negative; FH of RA;**

| **Paper** | **Study Design** | | | **Cohort** | **Autoantibody** | | | **Arthralgia** | **Family History of RA**  **(% 1^st^ degree relative)** | **Imaging** | **Outcome (concordance between two outcomes in%)** |  |
| --- | --- | --- | --- | --- | --- | --- | --- | --- | --- | --- | --- | --- |
| **Cohort: CCP2 & Arthralgia** | | | | | | | | | | | |  |
| Bos et al. ^20^ | Prospective Cohort | | | Amsterdam, Netherlands | CCP2 | | | Yes (100%) | No | No | >1 swollen joint or ACR 1987 criteria (34%) + (75%) |  |
| STAPRA ^2^ | RCT | | | RCT | CCP2 >3 ULN or IgM RF & CCP2(all patients were CCP2 positive) | | | Yes (100%) | No | No | >1 swollen joint or 2010 ACR/EULAR criteria (93%) |  |
| Eloff et al. 2020^22^ | Prospective Cohort | | | Linkoping Sweden | CCP2 | | | Yes (100%) | No | No | >1 swollen joint |  |
| Ten Brinck et al. ^19^ | | Prospective Cohort | Leiden (2012-2015), Netherlands | | CCP2 | | | Yes (100%) | No | No | >1 swollen joint | |
| APPIPRA | RCT | | | RCT | CCP2 >3 ULN or IgM RF & CCP2 (all patients were CCP2 positive) | | | Yes (100%) | No | No | >3 swollen joints or 2010 ACR/EULAR criteria (100%) |  |
| Duqenne et al. ^18^ | Prospective Cohort | | | Leeds | CCP2 | | | Yes (100%) | No | No | >1 swollen joint |  |
| Van Beers Tas ^21^ | Prospective Cohort | | | Amsterdam | CCP2 | | | Yes (100%) | No | No | >1 swollen joint |  |
| Pratt et al. | Prospective Cohort | | | NEAC | CCP2 | | | Yes (100%) | No | No | >1 swollen joint |  |
| **Cohort: CCP2 & IgM-RF & Arthralgia** | | | | | | | | | | | | |
| Bos et al. ^20^ | | Prospective Cohort | Amsterdam, Netherlands | | CCP2 | | IgM-RF | Yes (100%) | No | No | >1 swollen joint or ACR 1987 criteria (34%) + (75%) | |
| Eloff et al. 2020^22^ | | Prospective Cohort | Linkoping Sweden | | CCP2 | | Agglutinating RF/IgM-RF | Yes (100%) | No | No | >1 swollen joint | |
| Ten Brinck et al. ^19^ | | Prospective Cohort | Leiden (2012-2015), Netherlands | | CCP2 | | IgM-RF | Yes (100%) | No | No | >1 swollen joint | |
| Erlandson et al.^23^ | | Prospective Cohort | Gothenburg Sweden | | CCP2 | | IgM-RF | Yes (100%) | No | No | RA by ACR/EULAR 2010 Criteria | |
| Van Beers Tas et al. ^21^ | | Prospective Cohort | Amsterdam | | CCP2 | | IgM-RF | Yes (100%) | No | No | >1 swollen joint or 2010 ACR/EULAR Criteria (86%) | |
| APPIPRA | | RCT | RCT | | CCP2 >3 ULN or IgM RF & CCP2 (all patients were CCP2 positive) | | IgM-RF | Yes (100%) | No | No | >3 swollen joints or RA by ACR/EULAR 2010 Criteria (100%) | |
| **Cohort: CCP2 & IgM-RF negative** | | | | | | | | | | | | |
| Bos et al. ^20^ | | Prospective Cohort | Amsterdam, Netherlands | | CCP2 | IgM-RF | | Yes (100%) | No | No | >1 swollen joint or ACR 1987 criteria (34%) + (75%) | |
| Eloff et al. 2020^22^ | | Prospective Cohort | Linkoping Sweden | | CCP2 | IgM-RF/CarP negative | | Yes (100%) | No | No | >1 swollen joint | |
| Van Beers Tas ^21^ | | Prospective Cohort | Amsterdam | | CCP2 | IgM-RF | | Yes (100%) | No | No | >1 swollen joint | |
| NEAC | | Prospective Cohort | Newcastle | | CCP2 | IgM-RF | | Yes (100%) | No | No | >1 swollen joint | |
| APIPPRA | | RCT | RCT | | CCP2  CCP2 >3 ULN or IgM RF & CCP2 (all patients were CCP2 positive | IgM-RF | | Yes (100%) | No | No | >3 swollen joints or 2010 ACR/EULAR criteria (100%) | |
| **Cohort: CCP2 or CCP3 & 1^st^ or 2^nd^ degree relative with RA** | | | | | | | | | | | | |
| Bemis et al. (SERA) ^17^ | | Prospective Cohort | USA | | CCP2 or CCP3 | | | No | Yes (100%) | No | >1 swollen joint | |
| Tanner et al.  (North America Indigenous) ^25^ | | Prospective Cohort | Canada | | CCP2 or CCP3 | | | No | Yes (75%) | No | 2010 ACR/EULAR Criteria | |
| Gilbert et al. (SCREEN-RA) ^24^ | | Prospective Cohort | Switzerland | | CCP2 or CCP3 | | | No | Yes (100%) | No | >1 swollen joint | |


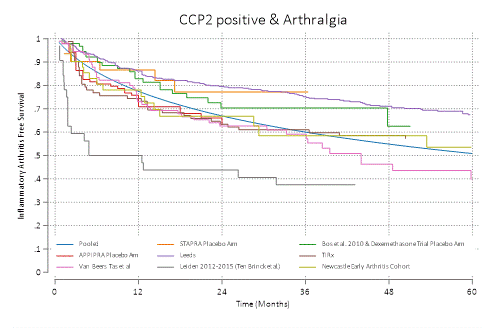


**D**

**C**

**B**

**A**

**Supplementary Figure 2 Individual Kaplan Maeir Survival Curves for Inflammatory Arthritis Free Survival in CCP2 positive cohorts, with pooled survival model superimposed; (A) CCP2 & Arthralgia; (B) CCP2 & Arthralgia & IgM-RF; (C) CCP2 & Arthralgia & IgM-RF negative; (D) CCP2 or CCP3 with 1^st^ or 2^nd^ degree relative with RA (FH of RA)**

**
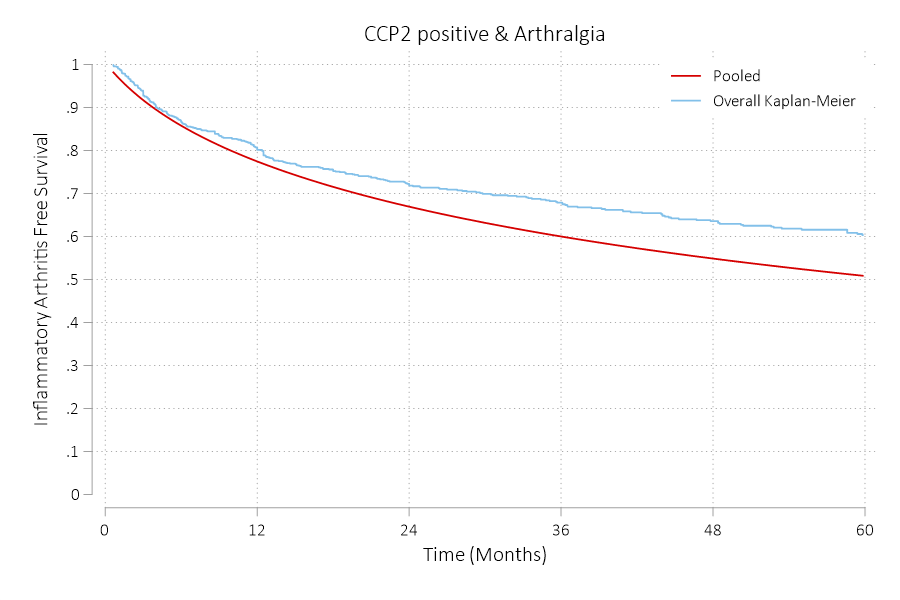
**

**B**

**A**

**C**

**D**

**Supplementary Figure 3. Overall Kaplan Maeir Curves for CCP (anti-citrullinated autoantibodies) positive individuals in combination with (A) CCP2 & arthralgia (B) CCP2 & arthralgia & IgM rheumatoid factor (IgM-RF) positive (C) CCP2 & arthralgia & IgM rheumatoid factor (IgM-RF) negative (D) CCP2 or CCP3 positive with 1^st^ or 2^nd^ degree relative with RA. Overall Kaplan-Maier Survival curves represents the total sum of individuals from each study (blue line), the pooled KM curve (red line) represents a pooled mixed effect model.**

**A**

**B**

**C**

**D**

**Supplementary Figure 4.** **Hazard function for CCP (anti-citrullinated autoantibodies) positive individuals in combination with (A) CCP2 & arthralgia (B) CCP2 & arthralgia & IgM rheumatoid factor (IgM-RF) positive (C) CCP2 & arthralgia & IgM rheumatoid factor (IgM-RF) negative (D) CCP2 or CCP3 positive with 1^st^ or 2^nd^ degree relative with RA.**

**Supplementary Results – Imaging Studies**

Studies which used imaging to enrol individuals into prospective cohorts most often used Magnetic Resonance Imaging (MRI) or Ultrasound in addition to autoantibody stratification and the presence of symptoms. Ultrasound most commonly assessed for power doppler (PD) or grayscale changes in joints, whereas MRI assessed for changes of osteitis, synovitis, and tenosynovitis.

We identified five studies, 3 RCT’s^3, 5, 28^ and 2 Prospective observational Cohort^26, 27^ , that examined imaging changes in CCP2 positive individuals with arthralgia (Supplementary Table 5).

**Supplementary Table 5. Eligible studies pooled according to imaging factors.**

| **Paper** | **Study Design** | **Cohort** | **Autoantibody** | | **Arthralgia** | **Joints examined** | **Ultrasound** | **MRI changes** | **Outcome (concordance between two outcomes in%)** |
| --- | --- | --- | --- | --- | --- | --- | --- | --- | --- |
| Cohort: CCP2 & Arthralgia & Ultrasound Power Doppler (maximum) == 0 | | | | | | | | | |
| Cope et al. (APPIPRA) | RCT | RCT | CCP2 >3 ULN | CCP2 & IgM-RF | Yes (100%) | Wrists, MCPJ, PIPJ, MTPJ (both limbs) | Yes | No | ≥3 swollen joints or RA by ACR/EULAR 2010 Criteria (100%) |
| Nam et al. 2016^26^ | Prospective Cohort | Leeds | CCP2 | | Yes (100%) | Wrists, MCPJ, PIPJ, MTPJ (both limbs) | Yes | No | ≥1 swollen joint |
| Cîrciumaru et al. 2024^27^ | Prospective Cohort | Sweden (Karolinska Risk-RA) | CCP2 | | Yes (100%) | Hands, Feet, Symptomatic joints, (both limbs) | Yes | No | ≥ 1 swollen joint |
| Cohort: CCP2 & Arthralgia & Ultrasound Power Doppler (maximum) == 1 | | | | | | | | | |
| APPIPRA^28^ | RCT | RCT | CCP2 >3 ULN | CCP2<3ULN & IgM-RF | Yes (100%) | Wrists, MCPJ, PIPJ, MTPJ (both limbs) | No | No | >3 swollen joints or RA by ACR/EULAR 2010 Criteria (100%) |
| Nam et al. 2016^26^ | Prospective Cohort | Leeds | CCP2 | | Yes (100%) | Wrists, MCPJ, PIPJ, MTPJ (both limbs) | No | No | ≥1 swollen joint |
| Cohort: CCP2 & Arthralgia & Ultrasound Power Doppler (maximum) == 2 | | | | | | | | | |
| Cope et al. APPIPRA^28^ | RCT | RCT | CCP2 >3 ULN | CCP2 & IgM-RF | Yes (100%) | Wrists, MCPJ, PIPJ, MTPJ (both limbs) | Yes | No | >3 swollen joints or RA by ACR/EULAR 2010 Criteria (100%) |
| Nam et al. 2016^26^ | Prospective Cohort | Leeds | CCP2 | | Yes (100%) | Wrists, MCPJ, PIPJ, MTPJ (both limbs) | Yes | No | >1 swollen joint |
| Cohort: CCP2 & Arthralgia & any MRI changes (osteitis, synovitis, tenosynovitis) | | | | | | | | | |
| Rech et al. (ARIAA)^5^ | RCT | RCT | CCP2 | | Yes (100%) | Wrist, MCPJ, PIPJ (dominant or most symptomatic hand) | No | Yes | >1 swollen joint |
| Krijbolder et al. (TREAT-EARLIER)^3^ | RCT | RCT | CCP2 | | Yes (100%) | Wrist, MCPJ, PIPJ and MTPJ (both limbs) | No | Yes | >2 swollen joints for >2 weeks or RA by ACR/EULAR 2010 Criteria |

**Supplementary Results - Ultrasound**

We were able to pool two studies which assessed power doppler changes in CCP2 positive individuals with joint symptoms (Supplementary Figure 5). The study sizes were small and assessed different total numbers of joints therefore any meaningful interpretation or modelling is limited, however there appeared to be a trend with increasing intensity of power doppler signal and cumulative incidence at 24 months. In individuals with PD signal of 1 there was a constant rate of progression to RA over the 24 months, whereas in the PD signal of 2, the progression to RA was higher within the first 12 months, after which the rate markedly reduced.

**A**

**B**

**Supplementary Figure 5: KM survival curves for CCP2 positive individuals with Arthralgia who have; A) 1; B) Power Doppler signal 2**

**Supplementary Results - MRI**

2 RCT’s used MRI to assess CCP2 positive individuals with joint symptoms for changes of osteitis, synovitis, or tenosynovitis and imaged either the most dominant/symptomatic hand joint or both hands and MTPJs Supplementary Figure 7. They reported progression rates of approximately 40%-50% at 12 months.

**Supplementary Figure 6: KM survival curves for CCP2 positive individuals with Arthralgia who have MRI changes of osteitis, tenosynovitis, or synovitis.**

Further studies, with homogenised imaging protocols and anatomical selection of joints would allow for more robust comparisons and accurate predictions, and analysis of the proportion of individuals with a second autoantibody would be instructive.

References

1. Bos, W. H., Dijkmans, B. A. C., Boers, M., van de Stadt, R. J. & van Schaardenburg, D. Effect of dexamethasone on autoantibody levels and arthritis development in patients with arthralgia: a randomised trial. *Ann Rheum Dis* 69, 571–574 (2010).

2. Van Boheemen, L. *et al.* Atorvastatin is unlikely to prevent rheumatoid arthritis in high risk individuals: results from the prematurely stopped STAtins to Prevent Rheumatoid Arthritis (STAPRA) trial. https://doi.org/10.1136/rmdopen-2021-001591 doi:10.1136/rmdopen-2021-001591.

3. Krijbolder, D. I. *et al.* Intervention with methotrexate in patients with arthralgia at risk of rheumatoid arthritis to reduce the development of persistent arthritis and its disease burden (TREAT EARLIER): a randomised, double-blind, placebo-controlled, proof-of-concept trial. *Lancet* 400, 283–294 (2022).

4. Cope, A. P. *et al.* Articles Abatacept in individuals at high risk of rheumatoid arthritis (APIPPRA): a randomised, double-blind, multicentre, parallel, placebo-controlled, phase 2b clinical trial. *www.thelancet.com* https://doi.org/10.1016/S0140-6736(23)02649-1 doi:10.1016/S0140-6736(23)02649-1.

5. Rech, J. *et al.* Abatacept inhibits inflammation and onset of rheumatoid arthritis in individuals at high risk (ARIAA): a randomised, international, multicentre, double-blind, placebo-controlled trial. *The Lancet* 403, 850–859 (2024).

6. Rantapää-Dahlqvist, S. *et al.* Antibodies Against Cyclic Citrullinated Peptide and IgA Rheumatoid Factor Predict the Development of Rheumatoid Arthritis. *Arthritis Rheum* 48, 2741–2749 (2003).

7. Jørgensen, K. T. *et al.* Cytokines, autoantibodies and viral antibodies in premorbid and postdiagnostic sera from patients with rheumatoid arthritis: Case-control study nested in a cohort of Norwegian blood donors. *Ann Rheum Dis* 67, 860–866 (2008).

8. Turesson, C. *et al.* Increased cartilage turnover and circulating autoantibodies in different subsets before the clinical onset of rheumatoid arthritis. *Ann Rheum Dis* 70, 520–522 (2011).

9. Shi, J. *et al.* Anti-carbamylated protein (anti-CarP) antibodies precede the onset of rheumatoid arthritis. *Ann Rheum Dis* 73, 780–783 (2014).

10. Arkema, E. V. *et al.* Anti-citrullinated peptide autoantibodies, human leukocyte antigen shared epitope and risk of future rheumatoid arthritis: A nested case-control study. *Arthritis Res Ther* 15, (2013).

11. Gan, R. W. *et al.* Anti-carbamylated protein antibodies are present prior to rheumatoid arthritis and are associated with its future diagnosis. *Journal of Rheumatology* 42, 572–579 (2015).

12. Brink, M. *et al.* Anti-carbamylated protein antibodies in the pre-symptomatic phase of rheumatoid arthritis, their relationship with multiple anti-citrulline peptide antibodies and association with radiological damage. *Arthritis Res Ther* 17, (2015).

13. Sundström, B., Johansson, I. & Rantapää-Dahlqvist, S. Interaction between dietary sodium and smoking increases the risk for rheumatoid arthritis: Results from a nested case-control study. *Rheumatology (United Kingdom)* 54, 487–493 (2015).

14. Fisher, B. A. *et al.* Smoking, Porphyromonas gingivalis and the immune response to citrullinated autoantigens before the clinical onset of rheumatoid arthritis in a Southern European nested case-control study Clinical rheumatology and osteoporosis. *BMC Musculoskelet Disord* 16, (2015).

15. Kelmenson, L. B. *et al.* Timing of Elevations of Autoantibody Isotypes Prior to Diagnosis of Rheumatoid Arthritis. *Arthritis and Rheumatology* 72, 251–261 (2020).

16. Mikuls, T. R. *et al.* Autoantibodies to Malondialdehyde–Acetaldehyde Are Detected Prior to Rheumatoid Arthritis Diagnosis and After Other Disease Specific Autoantibodies. *Arthritis and Rheumatology* 72, 2025–2029 (2020).

17. Bemis, E. A. *et al.* Factors associated with progression to inflammatory arthritis in first-degree relatives of individuals with RA following autoantibody positive screening in a non-clinical setting. *Ann Rheum Dis* 80, 154–161 (2021).

18. Ponchel, F. *et al.* Added value of multiple autoantibody testing for predicting progression to inflammatory arthritis in at-risk individuals. *RMD Open* 8, e002512 (2022).

19. ten Brinck, R. M. *et al.* The risk of individual autoantibodies, autoantibody combinations and levels for arthritis development in clinically suspect arthralgia. *Rheumatology (United Kingdom)* 56, 2145–2153 (2017).

20. Bos, W. H. *et al.* Arthritis development in patients with arthralgia is strongly associated with anti-citrullinated protein antibody status: A prospective cohort study. *Ann Rheum Dis* 69, 490–494 (2010).

21. van Beers-Tas, M. H. *et al.* The value of joint ultrasonography in predicting arthritis in seropositive patients with arthralgia: a prospective cohort study. *Arthritis Res Ther* 20, 279 (2018).

22. Eloff, E. *et al.* Autoantibodies are major predictors of arthritis development in patients with anti-citrullinated protein antibodies and musculoskeletal pain. *Scand J Rheumatol* 50, 189–197 (2021).

23. Erlandsson, M. C., Turkkila, M., Pullerits, R. & Bokarewa, M. I. Survivin measurement improves clinical prediction of transition from arthralgia to RA-Biomarkers to improve clinical sensitivity of transition from Arthralgia to RA. *Front Med (Lausanne)* 5, 219 (2018).

24. Gilbert, B. T. P. *et al.* Cohort profile: SCREEN-RA: design, methods and perspectives of a Swiss cohort study of first-degree relatives of patients with rheumatoid arthritis. *BMJ Open* 11, (2021).

25. Tanner, S. *et al.* A Prospective Study of the Development of Inflammatory Arthritis in the Family Members of Indigenous North American People With Rheumatoid Arthritis. *Arthritis and Rheumatology* 71, 1494–1503 (2019).

26. Nam, J. L. *et al.* Ultrasound findings predict progression to inflammatory arthritis in anti-CCP antibody-positive patients without clinical synovitis. *Ann Rheum Dis* 75, 2060–2067 (2016).

27. Cîrciumaru, A. *et al.* Identification of early risk factors for anti-citrullinated-protein-antibody positive rheumatoid arthritis—a prospective cohort study. *Rheumatology (Oxford)* 63, 3164 (2024).

28. Cope, A. P. *et al.* Abatacept in individuals at high risk of rheumatoid arthritis (APIPPRA): a randomised, double-blind, multicentre, parallel, placebo-controlled, phase 2b clinical trial. *The Lancet* 403, 838–849 (2024).
